# Evolution of invasive traits in nonindigenous species: increased survival and faster growth in invasive populations of rusty crayfish (*Orconectes rusticus*)

**DOI:** 10.1111/eva.12198

**Published:** 2014-08-27

**Authors:** Lindsey W Sargent, David M Lodge

**Affiliations:** 1 Department of Biological Sciences, University of Notre DameNotre Dame, IN, USA; 2 Department of Biological Sciences and Environmental Change Initiative, University of Notre DameNotre Dame, IN, USA

**Keywords:** adaptation, antipredator behavior, fish, food quality, growth rate, invasive species, mortality, predator

## Abstract

The importance of evolution in enhancing the invasiveness of species is not well understood, especially in animals. To evaluate evolution in crayfish invasions, we tested for differences in growth rate, survival, and response to predators between native and invaded range populations of rusty crayfish (*Orconectes rusticus*). We hypothesized that low conspecific densities during introductions into lakes would select for increased investment in growth and reproduction in invasive populations. We reared crayfish from both ranges in common garden experiments in lakes and mesocosms, the latter in which we also included treatments of predatory fish presence and food quality. In both lake and mesocosm experiments, *O. rusticus* from invasive populations had significantly faster growth rates and higher survival than individuals from the native range, especially in mesocosms where fish were present. There was no influence of within-range collection location on growth rate. Egg size was similar between ranges and did not affect crayfish growth. Our results, therefore, suggest that growth rate, which previous work has shown contributes to strong community-level impacts of this invasive species, has diverged since *O. rusticus* was introduced to the invaded range. This result highlights the need to consider evolutionary dynamics in invasive species mitigation strategies.

## Introduction

Evolution in nonindigenous populations contributes to the success and harmful impacts of some invasive species (Siemann and Rogers 2001; Handley et al. 2011), but how often this phenomenon occurs, especially for animals, is inadequately understood. Nonindigenous species can dramatically change biotic communities and ecosystem processes, sometimes causing extensive ecological or economic harm (Sala et al. 2000; Keller et al. 2009; Butchart et al. 2010). However, only a small percentage of nonindigenous species have strong community level impacts (Ricciardi and Kipp 2008). *r*-selected life history traits such as rapid growth and high fecundity are common among many of those nonindigenous species that have strong impacts (Sakai et al. 2001; van Kleunen et al. 2010; Lamarque et al. 2011), and characteristics of the environment and biotic community within the introduced range are also often important for invasion success (Catford et al. 2009). A subset of introduced species already possess *r*-selected life history traits upon arrival in a novel habitat, but other species may evolve toward these traits in response to selection in the invaded range.

Theory suggests that populations with lower conspecific densities should have greater *r*-selection (Lewontin 1965), and recent empirical data support this theory. For example, cane toads (*Bufo marinus* Linnaeus) from populations on an expanding range edge grow more rapidly than those from longer established populations when raised in common conditions and, therefore, reach reproductive maturity more quickly (Phillips 2009). In addition, a recent review of trait evolution in nonindigenous populations reveals that some invasive populations have evolved faster growth, wide environmental tolerance, shorter generation time, and increased reproductive capability in the invaded range (Whitney and Gabler 2008). However, few studies have tested for differences in invasive traits between populations using reciprocal transplant or common garden experiments (as opposed to comparative field observations), and almost all of these studies focus on introduced plants (but see Koskinen et al. 2002; Lee et al. 2003; Phillips 2009). Therefore, it remains unclear how often invasive traits evolve in nonindigenous animal populations. Though evolution within the invasive range may alter the impact of nonindigenous species, evolutionary potential is rarely included in risk assessments and policy decisions involving species introductions (Whitney and Gabler 2008).

To evaluate the likelihood of evolution influencing crayfish invasiveness, we conducted a series of common garden experiments to test whether differences existed in growth rate, survival, and response to predators in young of year (YOY) rusty crayfish (*Orconectes rusticus* Girard) from native and invasive populations. *O. rusticus* is one of many species of crayfish that have been introduced globally (Lodge et al. 2012). *O. rusticus*, in particular, cause major community level impacts in their invaded range. *O. rusticus* is native to streams in the Ohio River drainage in Ohio, Indiana, and Kentucky and was introduced by anglers to northern Wisconsin and Michigan lakes in the mid 1960s as well as to Illinois, Minnesota, Ontario (Canada), the Laurentian Great Lakes, and portions of 11 other states (Olden et al. 2006; Peters et al. 2014).

For this study, we focused on comparing well-studied invasive *O. rusticus* populations from northern Wisconsin to lesser studied native populations from the Ohio River drainage. Where *O. rusticus* has become abundant in Wisconsin and Michigan lakes, it has displaced resident crayfishes, reduced the abundance and richness of macrophytes and macroinvertebrates, and caused declines in the abundance of panfish (*Lepomis* spp.) (Capelli 1982; Lodge et al. 1994; Wilson et al. 2004; Olden et al. 2006). Faster growth of *O. rusticus* also contributes to the displacement of resident crayfishes (Hill et al. 1993; Garvey et al. 1994). In addition, *O. rusticus* reaches higher densities than other crayfishes in this region, which contributes to its strong impacts (Wilson et al. 2004). To our knowledge, the community level impacts of *O. rusticus* in the native range have not been investigated. Pintor and Sih (2009) found that *O. rusticus* from an invasive population grew more rapidly than *O. rusticus* from a native population when competing with congeners in mesocosms. However, because adult crayfish collected from the field were used in this study, it is unclear whether this result was due to evolution or to environmental differences between the two collection locations. Here, we use experiments to test for divergence in *r*-selected traits, specifically YOY growth rate and survival, in invasive *O. rusticus* populations in Wisconsin.

To determine whether there are widespread growth rate and survival differences between *O. rusticus* from native and invasive populations, we first reared crayfish from both ranges in enclosures in three lakes within the invaded range in summer 2011. We selected lakes with different abundances of predatory fish and macroinvertebrate prey to determine whether differences existed in growth rate or survival among different invasive range environments. Then in summer 2012, to provide evidence for the hypothesis that there is a genetic basis for the differences we observed, we reared crayfish in mesocosms where we controlled temperature and varied the presence of predatory fish and food quality to determine which factors were important in controlling *O. rusticus* growth rate and survival. Previous research indicates that predatory fish can reduce crayfish feeding activity (Stein and Magnuson 1976; Hill and Lodge 1995) and growth (Hill and Lodge 1999). We hypothesized that crayfish from the invaded range would respond less (i.e. smaller reduction in feeding activity) to predatory fish than those from the native range because there is likely to be a greater fitness benefit to allocating time to feeding (growth) within the invaded range. In addition, food quality (Hill et al. 1993) and temperature (Mundahl and Benton 1990) are important for crayfish growth. It is possible that rapid growth of invaded range crayfish can only be achieved in locations with abundant, high quality food resources. Our study is the first to test whether food quality and predator abundance have different effects on *O. rusticus* from native and invasive populations. Finally, we investigated the potential influence of maternal effects on results by testing for the effects of clutch and initial egg weight. Our study examines whether there are growth differences between replicated populations of rusty crayfish from the invaded and native range, and whether the observed differences have an environmental or genetic basis.

## Methods

### Lake common garden experiment

To test whether differences exist in growth rate and survival between native and invasive *O. rusticus*, we reared YOY crayfish from native and invasive populations in enclosures in invaded range lakes in summer 2011. We hand collected berried females (females with eggs attached to their abdomen) from the Great Miami (39°56′N, 83°44′W and 39°56′N, 83°42′W) and Little Miami (38°54′N, 83°34′W) river drainages in the native range in Ohio, USA and from High Lake (46°08′N, 89°32′W), Big Lake (46°11′N, 89°26′W), and Papoose Lake (46°10′N, 89°48′W) in the invaded range in Wisconsin, USA. Because temperatures are warmer in the native range than in the invaded range, *O. rusticus* females extrude eggs earlier in the native range. We collected females from streams in the native range in late April and from lakes in the invaded range in late May. Each female was placed in an individual container (18 × 18 cm) in the laboratory with constantly aerated well water, a shelter constructed from a polyvinyl chloride pipe, and gravel substrate. Eggs hatched, and young became independent from females 3–4 weeks after collection. Females were removed from containers once young were independent. YOY were fed a combination of spirulina disks and shrimp pellets *ad libitum* while in the laboratory. YOY were placed in lakes 1–2 weeks after they became independent from females (in late May for native range YOY and early July for invaded range YOY). All YOY were removed from lakes on September 9th after native range crayfish were in lakes for 15 weeks and invaded range crayfish were in lakes for 10 weeks.

Within lakes, crayfish were each housed in an individual clear plastic container (18 × 18 × 12.7 cm) with large rectangular holes (14 × 8 cm) cut into each side and replaced with window screen. Screened sides prevented crayfish escape, but allowed crayfish to be in contact with the physical and chemical lake environment and to receive visual cues from predators. Four to six grams of natural woody debris (sticks) were added to each container and four stones were glued to the bottom to increase container weight and provide shelter. Containers were placed between 0.25 and 0.5 m depth at one of two sites (sites were 50–100 m apart) in each of three lakes, Big Lake, High Lake, and Papoose Lake in Wisconsin, USA. Two sites were used in each lake to hedge against total loss of crayfish in the case of disturbance by humans, other animals, or storms. Each site contained 1 YOY from each brood (13 invasive females and 13 native females), so that there were a total of 26 YOY housed at each site and 52 YOY housed in each lake.

Lakes were chosen based on different invasion histories. Papoose Lake had high densities of *O. rusticus* (35 *O. rusticus* per trap in 2011), and therefore we expected this lake to have reduced macrophyte, macroinvertebrate, and panfish populations resulting from *O. rusticus* impacts (Wilson et al. 2004). High Lake had low densities of *O. rusticus* (4 *O. rusticus* per trap in 2011), and Big Lake had moderate densities of *O. rusticus* (19 *O. rusticus* per trap in 2010).

Total length of YOY was measured every 6–8 days. Growth rate was calculated as the difference between initial and final length divided by days in the experiment. Mortalities that occurred within the first 3 weeks of the experiment were replaced with individuals from the same brood that were housed in the laboratory with the same husbandry and conditions as provided after hatching.

Data from a previous preliminary experiment in Big Lake indicated that Big Lake YOY grown in containers (and fed only though natural colonization of the containers by macroinvertebrates) were equal in length to those YOY growing outside of containers at the end of the summer. Therefore, we did not add food to the experimental containers. Containers in all three lakes were quickly colonized by macroinvertebrates which provided food for crayfish. To provide an index of any differences in food availability among lakes, six containers without crayfish were placed in each lake in June. Macroinvertebrates were removed from these containers in August and preserved in 70% ethanol. We compared the ash free dry mass of these macroinvertebrates among lakes.

We also assessed temperature and the abundance of predatory fishes in each lake because they might affect crayfish growth rates. Hourly temperature was recorded at each site using temperature loggers (Onset Computer Corporation). Predatory fish abundance was assessed in each lake using three fyke nets set for one night at the end of June and one night at the end of July. Fyke nets were set within 50 m of crayfish sites, and thus were intended to measure fish activity at those specific locations.

### Mesocosm common garden experiment

To examine whether differences in growth rate and survival observed between YOY crayfish from native and invasive populations could be genetically based, and to identify important factors influencing growth rate and survival, we raised crayfish in common conditions in mesocosms in summer 2012. We used a 2 × 2 × 2 factorial design to examine the effects of range, predators, and food quality on the growth rate, survival, and behavior of rusty crayfish. As in the 2010 lake experiments, berried females were collected earlier from native range locations due to differences in reproductive timing between the two ranges. We hand collected berried females in early April from the Little Miami (38°54′N, 83°34′W and 39°47′N, 83°51′W) and Scioto River (40°00′N, 83°23′W) drainages in Ohio, USA. In early May, we collected berried females from High Lake (46°08′N, 89°32′W), Big Lake (46°11′N, 89°26′W), and Papoose Lake (46°10′N, 89°48′W) in Wisconsin, USA. Housing of females in the laboratory and all other husbandry practices were the same as for lake experiment unless specified below. YOY from the native range were placed in experimental mesocosms in late May, and YOY from the invaded range were placed in experimental mesocosms in late June. While native and invasive YOY were placed in mesocosms at different times during the summer, we controlled temperature and food availability so that all crayfish experienced the same environmental conditions throughout the experiment (as described below).

Within each mesocosm, ten invaded range and ten native range YOY crayfish were each housed individually in a clear plastic container with screened sides (identical to those used in lake experiments), so that the growth of each crayfish was independent and was not affected by the other crayfish in the mesocosm. Two stones were glued to one side of the bottom of the container to provide shelter for crayfish. On the opposite side, we attached a small nylon nut and bolt which held disks of prepared food (described below) securely in place. Crayfish, therefore, had to choose between feeding and hiding. Total length of crayfish was measured once every 7 days, and crayfish were removed from the experiment after 7 weeks. We replaced mortalities that occurred within the first 2 weeks of the experiment with crayfish from the same range and, if possible, the same brood. Replacement crayfish were housed in the laboratory with the same husbandry and conditions as provided after hatching.

Mesocosms consisted of 416 L plastic tanks with flow-through, aerated well-water, and were located in a wooded area on the shore of Trout Lake (Wisconsin, USA), under a suspended tarp to reduce light, falling debris, and heat load. There were 12 mesocosms in total, with 20 YOY *O. rusticus* in individual containers (10 invasive and 10 native) reared in each mesocosm. Temperature was maintained in each mesocosm by a 300 W heater, and each mesocosm was aerated to maintain high dissolved oxygen (8–10 mg/L) and uniform temperature. Hourly temperature was recorded in each mesocosm using temperature loggers (Onset Computer Corporation; mean temperature = 18.1°C, summertime range = 10–25°C). These temperatures are cooler than summer invaded range lake temperatures (mean temperature was 24.3°C in lake epilimnia during the first 7 weeks of crayfish growth in 2011); however, we were only able to heat well water to this extent in early summer, and needed to keep temperatures consistent later in the summer so that invaded range crayfish experienced the same conditions as native range crayfish. We tested whether temperature was different during native and invaded range crayfish growth periods using anova of average weekly temperature in each mesocosm. To examine whether predators had an effect on growth, six of the twelve mesocosms contained predatory fish (three bluegill, *Lepomis macrochirus* Rafinesque, and three smallmouth bass, *Micropterus dolomieu* Lacépède). Bluegill ranged in size from 9.5 to 13 cm total length during the experiment, and smallmouth bass ranged in size from 10.5 to 14.5 cm total length. Fish were fed *O. rusticus* (three per mesocosm) once per week and earthworms (*Lumbricus terrestris* Linnaeus) twice per week for the duration of the experiment. Fish readily consumed both food types. Bluegill and smallmouth bass are common in both the native and invaded range of *O. rusticus* (Boschung et al. 1983).

To examine the effect of food quality of crayfish growth, half of the crayfish in each mesocosm were fed high quality food and half were fed low quality food, which we created by mixing 500 mL of plant and animal matter with 20 g of sodium alginate and 750 mL of water. Using methods similar to those used by Cronin et al. (2002), we solidified food by pouring dissolved calcium chloride (14 g calcium chloride in 500 mL water) over a thin sheet of this food mixture. Food was cut into 2.5–5 g squares and secured in each container weekly with the nylon nut and bolt. Except for a few of the largest crayfish at the end of the experiment, some food remained in each container at the end of each week, so the amount of food was *ad libitum*. High quality food consisted of 40% macrophytes (*Potamogeton amplifolius* Tuck, *Potamogeton richardsonii* (Bennett) Rydberg, and *Sagittaria graminea* Michaux) and 60% animal matter (earthworms and bluegill filets). Low quality food was made from the same organisms, but contained 80% macrophytes and 20% animal matter. All food was frozen after it was made, and thawed within 1 day of placing it in the experiment. Native and invasive crayfish were fed from the same batch of food during the same week of growth.

### Statistical analyses

For the lake common garden experiment, we used anova to examine the effects of range (native or invasive) and lake on growth rate (mm/day). We also included initial length of YOY and maternal identity (clutch) as covariates to account for potential effects of these variables. Only crayfish that survived for the entirety of the experiment were used in the growth analysis. We also used anova to examine the effect of the collection location within each range on growth. For this analysis, we ran one anova for native range crayfish and one anova for invaded range crayfish, and also included the effect of lake (where YOY were housed) in each model. Initial length and clutch were not included in this second analysis because they were found to be unimportant in the first growth model. Because native and invaded range crayfish were placed in lakes at different times, we also conducted an analysis of weekly growth to better account for the varying effects of temperature and crayfish length throughout the summer. We used a linear mixed effects model to examine the effects of range, lake, length of crayfish (at the start of the week), and average temperature (during the one week growth period) on weekly crayfish growth. We included crayfish identity in this model as a random effect.

For the mesocosm common garden experiment, we used a linear mixed effects model to examine the effects on crayfish growth rate of range, fish, food quality, and all interactions between these variables. In addition, mesocosm nested within fish treatment was included as a random effect. We also included the effects of average temperature and initial length in the model to account for potential effects of these variables. Although initial length was found to be unimportant in the lake common garden analysis, we included it in this analysis because YOY in the mesocosm common garden were housed in the laboratory slightly longer; therefore, variance in initial size was greater and could have had a more substantial effect on growth. Because each crayfish was reared in a separate, individual container within the 12 mesocosms and was provided with its own food, the growth of each crayfish was independent and was not affected by the growth of other crayfish in the same mesocosm. Therefore, we analyzed the effects of range, food quality, temperature, and initial length at the individual crayfish level, and controlled for the influence of the mesocosm by including it as a random effect. On the other hand, fish treatment was applied at the mesocosm level, and thus we nested the effect of fish within mesocosm in the analysis, so that we conducted this analysis at the mesocosm level. We did not test for the effect of clutch in this analysis because YOY from females were not evenly divided between treatments as in the lake experiment. YOY used in this analysis came from 53 different clutches, with an average ± standard error (SE) of 3.75 ± 0.26 crayfish per clutch. It is, therefore, unlikely that the genotype of any one parent would drive growth rate trends. We included all crayfish that survived for at least 30 days in this analysis, so that we could increase our sample size while allowing sufficient time for crayfish to grow. There was no significant effect of survival time (30–50 days) on growth rate (invasive: *P* = 0.84, *r*^2^ = 0.0005; native: *P* = 0.57, *r*^2^ = 0.006). As for the lake experiment, we tested the effect of collection location within each range on growth rate. For this analysis, we used separate linear mixed models for crayfish from the native and invaded range and included the effects of fish treatment, average temperature, and initial length as fixed effects as well as mesocosm nested within fish treatment as a random effect. Food quality was not included because it was found to be unimportant in the first growth model.

For both the lake and mesocosm experiments, we used Cox Proportional Hazards Models to test the effect of range on YOY survival. We included lake as a fixed effect in the lake experiment model and predatory fish treatment and food quality as fixed effects in the mesocosm experiment model. We also included mesocosm nested within fish treatment as a random effect in the mesocosm experiment model.

To examine whether maternal investment was important for growth rate differences between native and invaded range crayfish, we compared egg mass between native and invaded range females in spring 2012. We obtained blotted wet weight for five to nine eggs from each of six females from the native range and nine females from the invaded range. Because female size may also affect egg mass, we analyzed these data using ancova to test the effects of range (native or invasive) and maternal carapace length on egg weight. We found that maternal carapace length was an important predictor of egg mass, and therefore could use maternal carapace length as an index of egg size for those crayfish grown in the common garden. We could not directly measure egg size for crayfish used in the experiment because removal of eggs from females causes egg mortality. We used ancova to test the effects of range, maternal carapace length (as a index of egg size), and their interaction on the initial and final length of YOY, and a linear model to examine how range, maternal carapace length, and their interaction influenced YOY growth rate. We also included fish treatment and average temperature as fixed effects in the linear model because these were important factors controlling growth rate. To examine how maternal carapace length (as a proxy for egg size) influenced YOY survival, we added maternal carapace length to the Cox Proportional Hazards Model for the mesocosm experiment.

In the mesocosm experiment, we also tested whether crayfish behavior differed between fish treatments by recording the location of each YOY when containers were opened once a week to measure crayfish and replace food. Starting in the fourth week of crayfish growth, we recorded the crayfish as ‘in shelter’ if it was under or motionless next to the rocks or screened sides of the container and ‘out of shelter’ if it was found away from the rocks and screen. We quantified the percentage of observations that were classified as ‘out of shelter’ for all native and invaded range crayfish in each mesocosm. We tested the effects of range and fish treatment on the percent of observations out of shelter in a mixed effects model with mesocosm included as a random effect.

## Results

### Lake common garden experiment

**Figure 1:**
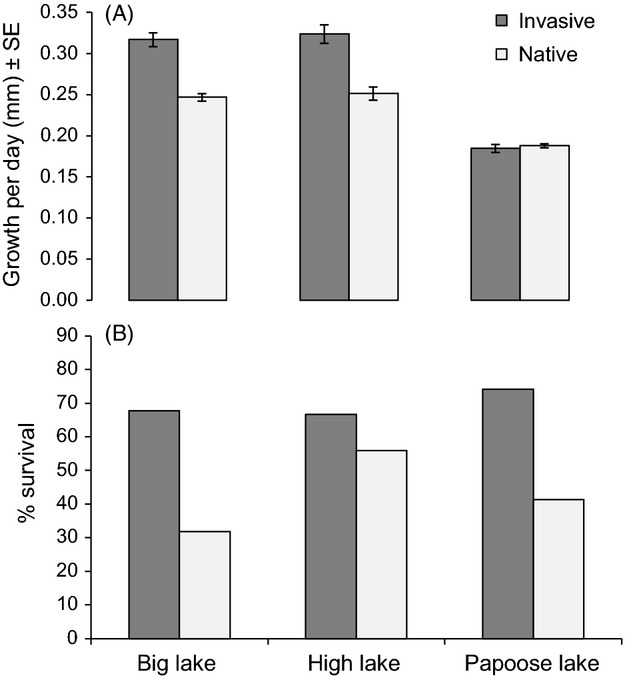
(A) Growth rate of *O. rusticus* from native and invasive range populations in lake common gardens. (B) Percent survival of native and invasive range crayfish over the course of the lake common garden experiment.

Over the course of the summer, *O. rusticus* from invasive populations grew more rapidly than *O. rusticus* from native populations (*F*_1,83_ = 22.13, *P* = 0.0033); lake (*F*_2,82_ = 73.87 *P* < 0.0001) and the interaction between lake and range also significantly affected growth rate (*F*_2,82_ = 8.56, *P* = 0.0175; Fig. 1A). Crayfish from invasive populations grew about 20% faster than crayfish from native populations in Big Lake and High Lake, but growth rates were similar between native and invaded range crayfish and about 30% slower in Papoose Lake (Fig. 1A). There was no significant effect of clutch or initial length on growth rate, or any other significant interactions between range, lake, clutch, or initial length (*P* > 0.4; Table S1).

We tested for differences in temperature between lakes to determine if temperature could cause differences in YOY growth rate observed between lakes. Temperature did not differ greatly between lakes. Average temperature in Big Lake (24.5°C) was very similar to that recorded in Papoose Lake (24.4°C) and High Lake (24.1°C) (see details in Fig. S1).

In contrast to temperature, we found substantial differences in fish and invertebrate abundance between lakes. Predatory fish species collected in fyke nets included bluegill, pumpkinseed (*L. gibbosus* Linnaeus), smallmouth bass, largemouth bass (*M. salmoides* Lacépède), rock bass (*Ambloplites rupestris* Rafinesque), and yellow perch (*Perca flavescens* Mitchill). Predatory fish were most abundant in High Lake (397 fish per trap night) followed by Big Lake (33 fish per trap night) and then Papoose Lake (19 fish per trap night) (see details in Table S2). Thus, growth rate differences were not consistent with inhibition of feeding in the presence of predatory fishes. Differences in invertebrate colonization among lakes were consistent with differences in growth rate of crayfish among lakes: invertebrates colonizing containers were most abundant in High Lake (0.066 ± 0.37 g ash free dry mass ± SE) followed by Big Lake (0.025 ± 0.006 g ash free dry mass ± SE), the two lakes where growth rates were highest, and then Papoose Lake (0.007 ± 0.001 g ash free dry mass ± SE) (see details in Fig. S2).

We also tested for within-range variation in growth rates to determine whether growth rate differences occur throughout the native and invaded range or were dependent on sampling location. While there was a significant impact of range on growth rate in the lake experiment, there was little within-range variation. For crayfish from the invaded range, lake of origin (population) was not a significant predictor of growth rate (*F*_2,52_ = 2.41, *P* = 0.1011), and there was no significant interaction between population and the lake where YOY were grown (*P* > 0.05). Similarly, for crayfish from the native range, river of origin (population) was not a significant predictor of growth rate (*F*_1,29_ = 0.57, *P* = 0.4589) and there was no interaction between population and the lake where YOY were grown (*P* > 0.05).

Crayfish from the invaded range also grew more rapidly than those from the native range in the weekly growth analysis (*F*_1,695_ = 7.74, *P* = 0.0069), and there was still a significant effect of lake on growth rate (*F*_1,695_ = 17.48, *P* < 0.0001). Temperature was also important for weekly growth (*F*_1,695_ = 17.48, *P* < 0.0001), and there was an interaction between length and lake indicating that there was no effect of length on growth rate in some lakes, but larger crayfish grew more slowly in other lakes (*F*_1,695_ = 8.53, *P* = 0.0002). There was also an interaction between range and length whereby larger native range crayfish grew more slowly, but there was no effect of length on growth rate in invaded range crayfish (*F*_1,695_ = 10.34, *P* = 0.0014).

In addition to differences in growth rate, we also tested for differences in survival. Native range crayfish were about 12% less likely to survive than invaded range crayfish within invaded range lakes (Cox Proportional Hazards Model coefficient = 1.1206, *z*_1,201_ = 3.056, *P* = 0.0022; Fig. 1B). Neither lake nor the interaction between lake and range were significant predictors of survival (*P* > 0.1).

### Mesocosm common garden experiment

**Figure 2:**
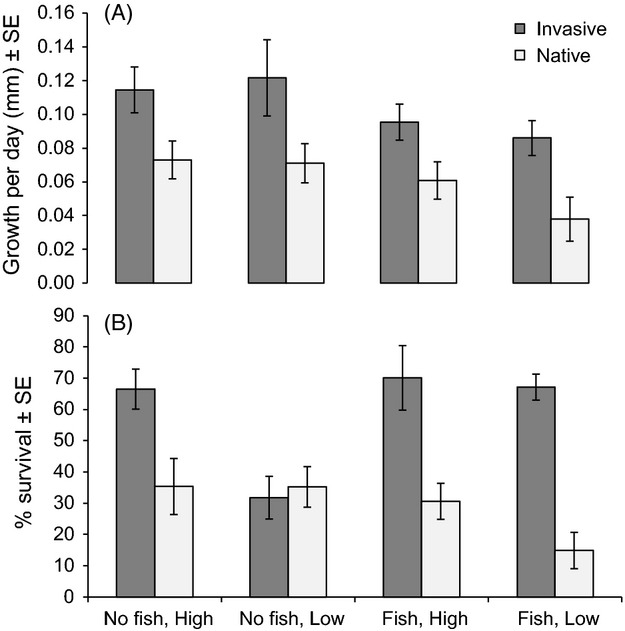
(A) Growth rate of *O. rusticus* from native and invasive range populations in mesocosm common gardens. (B) Percent survival of native and invasive range crayfish over the course of the mesocosm common garden experiment. Treatments include predatory fish absent or present × high or low quality food.

Growth results in the mesocosm experiment were similar to those from the lake experiment. Crayfish from invasive populations grew about 50% to 120% faster (depending on fish treatment and food quality) than crayfish from native populations (*F*_1,145_ = 21.41, *P* < 0.0001; Fig. 2A). We also found effects of fish presence and temperature on growth. Growth rates were about 40% lower in mesocosms with fish present (*F*_1,11_ = 5.48, *P* = 0.0412; Fig. 2A) and increased with temperature (*F*_1,145_ = 6.00, *P* < 0.0001). Despite our attempts to control temperature, crayfish from native populations experienced slightly warmer temperatures on average than crayfish from invasive populations (*F*_1,145_ = 6.01, *P* = 0.0154). Average temperature experienced by native range crayfish (±SE) was 18.4 ± 0.2°C, while average temperature experienced by invaded range crayfish (±SE) was 17.8 ± 0.2°C. The slower growing, native range crayfish experienced warmer temperatures; therefore, the positive effect of temperature on growth rate was weaker than the effect of range. There was no significant effect of food quality (*F*_1,145_ = 0.39, *P* = 0.5328) or initial length (*F*_1,145_ = 0.55, *P* = 0.4586) on crayfish growth. Average initial length ±SE of YOY at the start of the experiment (when placed in the mesocosms) was 13.9 ± 0.2 mm for crayfish from invasive populations and 13.2 ± 0.2 mm for crayfish from native populations. There was a significant interaction between initial length and range (*F*_1,145_ = 6.99, *P* = 0.0093), indicating that growth rate decreased with initial size in invaded range crayfish (*r*^2^ = 0.03), but increased with initial size in native range crayfish (*r*^2^ = 0.08). All other interactions between range, fish treatment, food quality and initial length were non-significant (*P* > 0.1; Table S3).

Also similar to the lake experiment, no significant effect existed of within-range lake or river of origin on growth rate for either invaded range or native range crayfish (*F*_1,92_ = 0.24, *P* = 0.7881, and *F*_1,52_ = 1.31, *P* = 0.2859, respectively). In addition, there were no significant interactions between within-range population and any other variable in the models (*P* > 0.1).

As in the lake experiment, crayfish from native populations were about 12% less likely to survive during the experiment than crayfish from invasive populations across treatments (Table 1; Fig. 2). In addition, crayfish that received low quality food were roughly 13% less likely to survive than crayfish that received high quality food (Table 1; Fig. 2B. Significant interactions existed between range, fish treatment, and food quality on crayfish survival (Table 1; Fig. 2B). Overall, within crayfish from the invaded range, individuals had the lowest survival when fish were absent and they received low quality food. Within crayfish from the native range, individuals had the lowest survival when fish were present and they received low quality food.

**Table 1:** Cox Proportional Hazards Model for crayfish survival in the mesocosm common garden experiment. A total of 333 crayfish were used in this analysis

Factor	Coefficient	*Z*	*P*
Range (native)	1.122	3.16	0.0016*
Fish (present)	0.106	0.25	0.8000
Food Quality (low)	1.128	3.18	0.0015*
Range*Fish	0.042	0.09	0.9300
Range*Food Quality	−1.178	−2.67	0.0075*
Fish*Food Quality	−0.988	−1.88	0.0600
Range*Fish*Food Quality	1.254	1.99	0.0470*

* *P* < 0.05.

### Maternal effects

**Figure 3:**
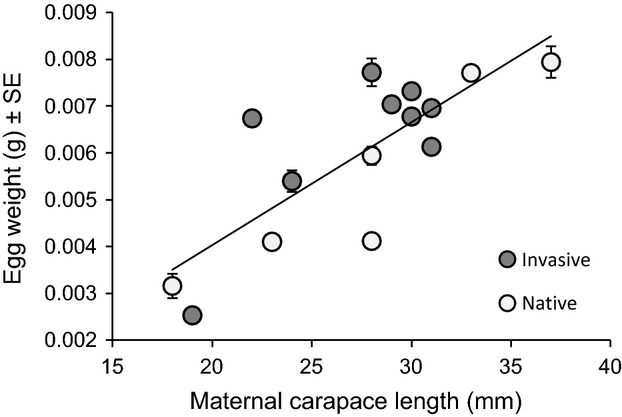
Relationship between maternal carapace length and egg weight in native and invasive range *O. rusticus*.

Overall, there was little evidence for significant effects of egg weight on growth rate or survival. There was no significant difference in egg weight between crayfish from native and invasive populations (*F*_1,11_ = 3.16, *P* = 0.1030; Fig. 3), and no interaction between range and maternal carapace length on egg weight (*F*_1,11_ = 0.01, *P* = 0.9250; Fig. 3), indicating that native and invaded range females of the same size produced eggs of the same size. However, larger females from both ranges produced significantly larger eggs than small females (*F*_1,11_ = 24.82, *P* = 0.0004; Fig. 3).

**Figure 4:**
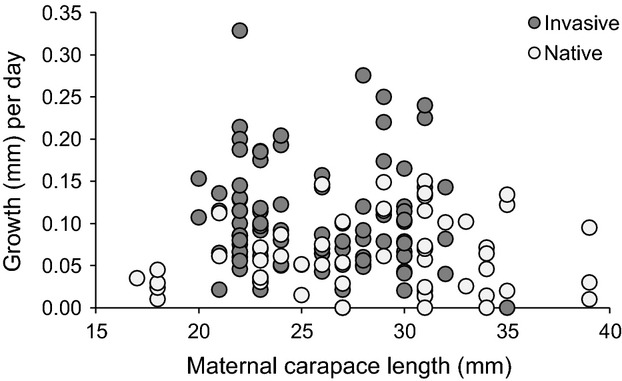
Relationship between maternal carapace length (as a proxy for egg size) and growth per day in native and invasive range *O. rusticus*.

Further, while larger females produced larger young, there was no effect of maternal size on growth rate or survival. At the beginning of the mesocosm experiment (when YOY were placed in mesocosms), there was a significant positive relationship between maternal carapace length (as a proxy for egg size) and carapace length of offspring (*F*_1,142_ = 33.21, *P* < 0.0001). There was also a significant interaction between maternal carapace length and range on offspring size (*F*_1,142_ = 5.26, *P* = 0.0233), indicating that maternal carapace length had a greater positive effect on invaded range offspring than native range offspring. At the end of the experiment, maternal carapace length still had a positive influence on offspring size (*F*_1,142_ = 6.32, *P* = 0.0131), but the interaction between maternal carapace length and range on offspring size was non-significant (*F*_1,142_ = 0.08, *P* = 0.7810). The relationship between maternal carapace length and offspring growth rate during the experiment was also non-significant (*F*_1,142_ < 0.01, *P* = 0.9823; Fig. 4), and there were no significant interactions between maternal carapace length and any other variable in the growth model (*P* > 0.1). Further, there was no significant effect of maternal carapace length on survival (coefficient = 0.0268, *z*_1,332_ = 0.29, *P* = 0.77) nor any significant interaction between maternal carapace length and any other variable in the Cox Proportional Hazards Model (P > 0.4). Overall, these results indicate that egg weight did not drive the observed differences in growth rate and survival between native and invaded range crayfish.

### Crayfish behavior

Crayfish behavior differed between fish treatments. Crayfish in mesocosms without fish were more likely to be found outside of shelter (*F*_1,23_ = 27.11, *P* < 0.0001). In addition, there was a non-significant trend suggesting that crayfish from native populations spent more time outside of shelter than crayfish from invasive populations (*F*_1,23_ = 3.24 *P* = 0.0878). Invaded range crayfish were found outside of shelter (±SE) 75 ± 5% of the time in mesocosms without fish and 52 ± 2% of the time in mesocosms with fish, and native range crayfish were found outside of shelter 86 ± 1% of the time in mesocosms without fish and 59 ± 7% of the time in mesocosms with fish. There was no interaction between range and fish treatment on behavior (*P* = 0.65).

## Discussion

### Growth rate differences

In both lake and mesocosm common garden experiments, invasive crayfish had faster growth rates than native crayfish. Data indicate that these growth rate differences were not due to differences in egg weight between the two ranges. Overall, these findings are consistent with evolution of faster growth rates within the invaded range. While larger females initially produced larger young (presumably because of the positive relationship between maternal carapace length and egg weight), there was no significant effect of maternal length on growth. In addition, in both lake and mesocosm experiments we found that within each range young collected as eggs from different lakes or streams had similar growth rates. These results provide further evidence that the observed growth rate differences are due to differences that characterize the ranges (native vs. invaded) rather than sampling locations within each range.

While our data are consistent with evolution of faster growth in invasive populations of *O. rusticus,* we cannot completely rule out the influence of maternal effects. However, maternal effects are less likely to control the differences we observed in growth rates than genetic differences because eggs from the largest females in our study were roughly 3× larger than the eggs from the smallest females, and we did not detect a significant effect of this difference in egg size on YOY growth rate (Fig. 4). Other research has found little influence of maternal effects on offspring quality in other decapods (Tropea et al. 2012; Swiney et al. 2013), or that maternal effects scale with female size (Sato and Suzuki 2010). However, there could potentially be other maternal effects such as differences in hormones or specific nutrients within eggs that could affect growth rate. We intentionally collected females from lakes with variable invertebrate prey availability, and there was no effect of within-range lake or river of origin on growth rate in either common garden experiment. We therefore expect that the differences in growth rates we observed were most likely genetically based.

Within the lake common garden experiment, we placed crayfish from the native range in the lakes earlier than crayfish from the invaded range because of differences in timing of reproduction. Growth differences, therefore, could have been due to differences in temperature and/or food availability during the initial weeks of YOY growth. However, because we were able to control the external environment including temperature and food availability in mesocosms, our results are consistent with a genetic basis for the observed differences in growth rates between native and invaded range crayfish. Lake experiments suggest that this phenomenon occurs not only in the laboratory, but also in natural environments.

Although we were not completely successful controlling temperature in the mesocosm experiment, the differences in temperature experienced by invasive and native range crayfish are not consistent with the differences in growth rate between populations (i.e., if temperature had been the primary driver of growth rates, the differences in growth rate would have been in the opposite direction from those we observed). Still, because crayfish were reared at different times in the mesocosm experiment, it is possible that an unmeasured factor affecting growth rate could have influenced our results. However, this is unlikely because we observed consistent differences in growth rate between native and invaded range crayfish across mesocosms where we varied important factors such temperature, food availability and predator presence. Eggs were also exposed to the environment within their lake or river of origin for a few weeks before collection. We also think this is less likely than genetic differences to be responsible for the observed differences in growth rate because we collected eggs from diverse environments within each range and the majority of egg development occurred in identical conditions in the laboratory.

Our results suggest that food availability differences among lakes were important for differences in crayfish growth rate. In the lake common garden experiment, the positive relationship between prey abundance and crayfish growth rate suggests that food availability was an important driver of growth rate differences; however, we found no effect of food quality on growth rate in the mesocosm experiment. It is possible that less food was available in the lake with the lowest invertebrate biomass (Papoose Lake) than we provided in the low quality food treatment of the mesocosm experiment (which still contained 20% animal matter). We expect that providing less food in the mesocosm experiment would have made food quality an important predictor of growth rate in this experiment as well.

Predatory fish presence, in contrast, was a significant predictor of crayfish growth rate in the mesocosm experiment but not in the lake experiment (i.e., the lake with the slowest growth had the lowest abundance of predatory fish). We expect that the effect of fish was more pronounced in the mesocosm experiment because fish were completely absent from some mesocosms but were present at different densities in all lakes. Behavioral data suggest that reduced growth rates associated with fish presence are likely due to the behavioral response of crayfish to fish. Results indicated that crayfish spent more time hiding (and therefore not consuming food) when fish were present. Together these data suggest that nonconsumptive effects of fish do reduce crayfish growth rates, but in natural systems, low densities of predators can have similar effects to high densities of predators.

The mesocosm experiment also revealed that initial length and temperature were important predictors of YOY growth rate. Invaded range crayfish were slightly larger on average than native range crayfish at the start of the mesocosm experiment (by an average of 0.7 mm carapace length), and there was a significant interaction between initial length and range on growth. Larger invaded range crayfish tended to grow slower over the course of the experiment, which is consistent with the well-documented pattern of declining growth rate with increasing size in many animals (Ricklefs 1967). Native range crayfish did not get as large as invaded range crayfish in the mesocosm study which may be why there was no decline in growth rate for these individuals and the largest individuals grew most rapidly. We also observed a negative relationship between native range crayfish length and growth rate in the lake study likely for the same reason. Native range crayfish were largest at the end of the lake study because they had a longer growing period. As observed in previous studies (e.g., Mundahl and Benton 1990), crayfish grew faster in warmer water, but this was clearly not sufficient to overshadow the differences between native and invasive populations.

While crayfish from northern Wisconsin grew more rapidly than those from the Ohio River drainage, it is unclear whether this would lead to larger young within the invaded range compared to those within the native range because of temperature differences between these two locations. Previous studies examining growth of YOY *O. rusticus* have found YOY carapace lengths ranging from 9 to 16.5 mm in September in northern Wisconsin (Lorman 1980) and YOY carapace lengths ranging from 8 to 17 mm in September in northern Kentucky (Prins 1968). In a preliminary study in 2010, we collected YOY crayfish from Big Lake in northern Wisconsin in August, which ranged from 9.5 to 15.5 mm carapace length. While these measurements are restricted to specific locations within each range, and not necessarily representative of growth rates throughout each range, they suggest that if there are differences in crayfish size between these two ranges, they are not large.

### Survival differences

Not only did native range crayfish have reduced growth, they also had reduced survival in both the lake and mesocosm common garden experiments. This could be a result of local adaptation of invasive *O. rusticus* populations to environmental conditions in the invaded range, especially if some characteristics in the mesocosm experiment more closely resembled lakes in northern Wisconsin than streams in the Ohio River drainage. We expect calcium concentration was lower in lakes and mesocosms than it is in Ohio streams, and flow also differs between these environment types. Differences in growth rates could also be attributed to local adaptation of the invasive population to environmental characteristics such as these. However, if the differences in growth rates observed between the two ranges were due to local adaptation to calcium concentration or flow rate, we would expect to see larger YOY at the end of the summer in the native range where temperatures are warmer.

In the mesocosm experiment, food quality and predatory fish presence had similar effects on native and invaded range crayfish growth, but these factors differentially influenced native and invaded range crayfish survival. Invaded range crayfish were more likely to survive than native range crayfish in all treatments except when food quality was low and no fish were present. Higher mortality within this group was unexpected, but may be due to the combination of rapid growth and low quality food, which could potentially lead to higher mortality due to unavailability of essential nutrients. In addition, crayfish in this group had the fastest growth rates on the low quality diet, and thus likely ate a greater quantity of low quality food than other crayfish. Therefore, secondary metabolites from macrophytes in the low quality diet could also be responsible for the observed increase in crayfish mortality within this group. Crayfish from the native range had the lowest survival when food quality was low and fish were present. This finding suggests a strong behavioral response of native range crayfish to fish that results in reduced feeding or increased energy expenditure. This response was not observed in invaded range crayfish. Because of high mortality, those crayfish that had the greatest behavioral response to fish may not have survived long enough to be included in the growth results, which may be why there is no interaction between range and fish presence apparent from the growth data. In addition, higher mortality of native range crayfish may also explain the trend that surviving native range crayfish spent more time outside of shelter than surviving invaded range crayfish. If predation pressure is similar between the two ranges, we expect it will be more beneficial for invaded range crayfish to favor feeding over predator avoidance because there should be a greater fitness benefit associated with fast growth in this range.

### Mechanisms leading to growth rate evolution in invasive populations

The finding that *O. rusticus* from invasive populations have faster growth rates than those from native populations was consistent with our expectations of how natural selection within the invaded range would alter this trait. Larger crayfish produce more eggs than small crayfish (Savolainen et al. 1997; Skurdal et al. 2011), so crayfish with faster growth have greater reproductive output. Life history theory predicts that optimal life-history strategies will differ between density-regulated and non-density-regulated populations, with higher fitness associated with high reproductive rates in non-density-regulated populations (Roughgarden 1971; Burton et al. 2010). Invasive populations are non-density-regulated in the early stages of an introduction. Some previous studies have found evidence for evolution of *r*-selected life history traits in invasive populations or during range expansion (Burton et al. 2010; Phillips et al. 2010; Flory et al. 2011); however, other studies have found no evidence for the evolution of these traits (e.g. Bossdorf et al. 2004; Cripps et al. 2009). We expect that there may be a more lasting effect of life history evolution in aquatic invasive species compared to most terrestrial species. Range edges, or locations with low conspecific densities, are scattered throughout the invaded range for most aquatic species. Lakes are insular environments and uncolonized lakes are spread throughout the invaded range; therefore, many aquatic invasive populations are serially introduced into locations with low conspecific densities. Thus, we hypothesize that compared to invaders in most terrestrial or marine environments, aquatic invaders will experience exponential growth more often, and there will be a stronger or longer lasting effect of *r*-selection in these populations.

Evolution of increased competitive ability (EICA) is another mechanism which could lead to evolution of rapid growth in invasive populations. EICA postulates that release from natural enemies such as predators and parasites allows nonindigenous species to allocate more resources toward growth (Keane and Crawley 2002; Inderjit and van der Putten 2010). However, because there are native congeners in northern Wisconsin lakes, there are many predators and parasites that readily consume or infect *O. rusticus*. Predatory fish are important in controlling *O. rusticus* populations (Roth et al. 2007) and high levels of parasitism by trematodes have been observed in some lakes (Roesler 2009). Therefore, we think this mechanism is less likely to be responsible for the higher growth rates observed in invasive *O. rusticus* than life history trait selection.

Hybridization may enhance the likelihood that nonindigenous populations will evolve invasive traits (Ellstrand and Schierenbeck 2000). Within the invaded range, *O. rusticus* hybridizes with a resident congener, *Orconectes propinquus* Girard (Perry et al. 2001)*. O. rusticus* is competitively superior, and hybrids produce offspring that are most likely to backcross with *O. rusticus* (Perry et al. 2001). It is unclear whether *O. propinquus* alleles remain in invasive *O. rusticus* (hybrid) populations over time (Perry et al. 2001). Since the early 1980s, no *O. propinquus* have been detected in any of the lakes where we collected *O. rusticus*, and *O. propinquus* has never been detected in Big Lake (Lodge unpublished data); therefore, we were not examining populations that hybridized recently. *O. propinquus* grow more slowly than *O. rusticus* (Hill et al. 1993), so rapidly growing invasive *O. rusticus* represent novel genotypes that are dissimilar from both parental populations. It is, however, possible that earlier hybridization and introgression provided increased additive genetic variance or created novel epistatic interactions that allowed *O. rusticus* to evolve faster growth rates in the invaded range.

### Community impacts of growth rate divergence

Rapid growth rates in invasive *O. rusticus* have had major community-level consequences. *Orconectes rusticus* has a greater impact on the ecological community than congeners, *O. virilis* Hagen and *O. propinquus*, and often causes declines in macrophyte and macroinvertebrate abundance and richness when replacing these species (Wilson et al. 2004). The ability of *O. rusticus* to replace *O. propinquus* has been attributed in part to its faster growth rate and ability to outcompete smaller individuals for shelter (Hill et al. 1993; Garvey et al. 1994; Hill and Lodge 1994). Faster growth also causes *O. rusticus* to escape predation from gape-limited fish more rapidly (Stein 1977). Understanding how often nonindigenous organisms evolve invasive traits is crucial for understanding the costs and consequences associated with introducing species to new locations.

### Implications for management of invasions

We recommend that the potential for populations to evolve increasingly invasive traits be considered when moving species to new locations. Even though a species may not be problematic in its native range, or may be unproblematic initially in a new location, traits such as rapid growth and high reproductive output that may increase ecological impacts can evolve within the invaded range. This may be especially likely to occur in populations which are serially introduced to insular environments such as aquatic organisms in lakes.

Risk assessments that do not include evolutionary potential may underestimate the likelihood of a species to cause ecological and economic harm. Species that are likely to hybridize with native species may also be especially likely to evolve in response to selection within the invaded range because of increased additive genetic variance in hybrids. Crayfish in North America are a prime example of organisms that are likely to evolve invasive traits when introduced to new locations because they live in patchy insular environments (lakes or stream drainages), and because they are likely to encounter native crayfishes with which hybridization may be possible. Especially when introduced to new locations within North America, crayfish are often exposed to closely-related, native species with which they are likely to hybridize (Perry et al. 2002). Seventy-five percent of the world's crayfish species are found within the United States (Lodge et al. 2000). Despite these problems, many states in the United States do not regulate the movement of crayfish or encourage voluntary practices to restrict moving crayfish, and many other states have legislation that only restricts moving certain species that are known to be problematic (Peters and Lodge 2009; Dresser and Swanson 2013). Our research suggests that invasive traits can evolve in nonindigenous crayfish populations, and this risk could be considered when weighing the costs and benefits of moving crayfish to new locations.

## Data archiving statement

Data underlying the main results of this study are available from the Dryad Digital Repository.

## Supporting Information

**Table S1.** anova table for growth within the lake experiment. **Table S2.** Fish species and numbers collected in Fyke nets in High Lake, Papoose Lake, and Big Lake in summer 2011. **Table S3.**anova table for growth within the mesocosm experiment. **Figure S1.** The cumulative number of degree days (°C × days) in Big Lake, High Lake, and Papoose Lake in summer 2011. **Figure S2.** Average quantity of invertebrates from different orders that colonized control containers (containers without crayfish) ±SE in High Lake, Big Lake and Papoose Lake in summer 2011.
