# Supplementary material for: Evolution of invasive traits in nonindigenous species: increased survival and faster growth in invasive populations of rusty crayfish (Orconectes rusticus)
Source: Evol Appl. 2014 Aug 27;7(8):949–61. doi: 10.1111/eva.12198 (PMC4211724; doi:10.1111/eva.12198)
Supplement: Supplementary file 1 [file eva0007-0949-sd1.docx]

**Supporting Information**

**Table S1** ANOVA table for growth within the lake experiment.

**Table S2** Fish species and numbers collected in Fyke nets in High Lake, Papoose Lake, and Big Lake in summer 2011.

**Table S3** ANOVA table for growth within the mesocosm experiment.

**Figure S1** The cumulative number of degree days (°C x days) in Big Lake, High Lake, and Papoose Lake in summer 2011.

**Figure S2** Average quantity of invertebrates from different orders that colonized control containers (containers without crayfish) ± SE in High Lake, Big Lake and Papoose Lake in summer 2011.

**Table S1** ANOVA table for growth rate within the lake experiment.

| Factor | df | F | P |
| --- | --- | --- | --- |
| Range | 1 | 22.13 | 0.0033* |
| Lake | 2 | 73.87 | <0.0001* |
| Initial Length | 1 | 0.79 | 0.4083 |
| Clutch | 21 | 0.65 | 0.7858 |
| Range*Lake | 1 | 8.56 | 0.0175* |
| Range*Initial Length | 1 | 0.06 | 0.8213 |
| Lake*Initial Length | 2 | 0.20 | 0.8248 |
| Lake*Clutch | 30 | 0.66 | 0.7929 |
| Initial Length*Clutch | 12 | 0.31 | 0.9597 |
| Range*Lake*Initial Length | 1 | <0.01 | 0.9466 |
| Lake*Initial Length*Clutch | 4 | 0.38 | 0.8167 |

**Table S2** Fish species and numbers collected in Fyke nets in High Lake, Papoose Lake, and Big Lake in summer 2011.

| Lake | Species | June Fyke Nets | July Fyke Nets |
| --- | --- | --- | --- |
| High Lake | Bluegill | 529 | 157 |
|  | Rock Bass | 27 | 23 |
|  | Pumpkinseed | 24 | 14 |
|  | Yellow Perch | 6 | 4 |
|  | Largemouth Bass | 2 | 5 |
|  | Smallmouth Bass | 1 | 1 |
| Papoose Lake | Bluegill | 3 | 0 |
|  | Rock Bass | 16 | 14 |
|  | Pumpkinseed | 1 | 0 |
|  | Yellow Perch | 0 | 1 |
|  | Largemouth Bass | 0 | 0 |
|  | Smallmouth Bass | 2 | 1 |
| Big Lake | Bluegill | 9 | 17 |
|  | Rock Bass | 12 | 7 |
|  | Pumpkinseed | 0 | 2 |
|  | Yellow Perch | 0 | 16 |
|  | Largemouth Bass | 0 | 0 |
|  | Smallmouth Bass | 1 | 1 |

Quantities represent the total number of each species collected from three Fyke nets.

**Table S3** ANOVA table for growth rate within the mesocosm experiment.

| Factor | df | F | P |
| --- | --- | --- | --- |
| Range | 1 | 21.41 | <0.0001* |
| Fish | 1 | 5.49 | 0.0412* |
| Food Quality | 1 | 0.39 | 0.5328 |
| Initial Length | 1 | 0.55 | 0.4586 |
| Temperature | 1 | 26.00 | <0.0001* |
| Range*Fish | 1 | 1.16 | 0.2846 |
| Range*Food Quality | 1 | 0.023 | 0.8787 |
| Fish*Food Quality | 1 | 1.67 | 0.1986 |
| Range*Initial Length | 1 | 6.99 | 0.0093* |
| Fish*Initial Length | 1 | 2.28 | 0.1340 |
| Food Quality*Initial Length | 1 | 0.03 | 0.8557 |
| Range*Fish*Food Quality | 1 | 0.06 | 0.8042 |
| Range*Fish*Initial Length | 1 | 1.54 | 0.2172 |
| Range*Food Quality*Initial Length | 1 | 0.83 | 0.3638 |
| Fish*Food Quality*Initial Length | 1 | 0.21 | 0.6513 |
| Range*Fish*Food Quality*Initial Length | 1 | 0.68 | 0.4107 |

**Figure S1** The cumulative number of degree days (°C x days) in Big Lake, High Lake, and Papoose Lake in summer 2011.

**Figure S2** Average quantity of invertebrates from different orders that colonized control containers (containers without crayfish) ± SE in High Lake, Big Lake and Papoose Lake in summer 2011.
